# PERSPECTIVE: organelle positioning as a principle of metabolic regulation and stress tolerance

**DOI:** 10.1111/tpj.70987

**Published:** 2026-06-25

**Authors:** Alisdair R. Fernie, Guillaume Decros, Jan Multhoff, Jana Sippel, Jan‐Ole Niemeier, Pedro Barreto, Arun Sampathkumar, Uwe Sonnewald, Markus Schwarzländer

**Affiliations:** ^1^ Max Planck Institute of Molecular Plant Physiology Am Mühlenberg 1 14476 Potsdam‐Golm Germany; ^2^ Institute of Plant Biology and Biotechnology (IBBP), University of Münster Schlossplatz 8 Münster 48143 Germany; ^3^ Division of Biochemistry, Department of Biology Fredrich‐Alexander‐University, Erlangen‐Nuremberg Staudtstrasse 5 91058 Erlangen Germany

**Keywords:** energy metabolism, organellar interaction, organellar dynamic movements, mitochondria, chloroplast

## Abstract

Plants have evolved elaborate acclimation strategies to withstand adverse environments, involving all levels of their function and organization. While macroscopic movements through differential growth that enable the physical re‐shaping and ‐positioning of organs have been a major field of study, the highly active dynamics of cell organelles within the immobile plant cell hold major unresolved questions. Here, we argue that the precise positioning of cell organelles is not only critical for cell division and development but also represents a fundamental principle in regulating cellular metabolism and underpinning effective responses to biotic and abiotic stresses. Although the positioning of individual organelles, such as nuclei and chloroplasts, and probably to a lesser extent, mitochondria, peroxisomes, endoplasmic reticulum tubules, Golgi bodies, and lipid droplets, is clearly controlled by developmental and environmental stimuli, the underlying mechanisms and functional significance remain unclear in many instances. We discuss steps required to improve our understanding of how intracellular positioning of organelles in general, and of chloroplasts and mitochondria in particular, contributes to metabolic regulation. Drawing on the recent discovery that three glycolytic enzymes can physically tether mitochondria and chloroplasts, we highlight approaches and concepts to establish how the spatial arrangement of organelles relative to one another underpins regulation of metabolism and stress responses.

## Significance Statement

Understanding how plant cells coordinate metabolism remains a major challenge, particularly at the level of intracellular organization. This perspective proposes that dynamic organelle positioning acts as a fundamental regulatory principle of metabolism, providing a conceptual and experimental framework to uncover how spatial organization shapes plant metabolic efficiency and stress tolerance.

## INTRODUCTION

**Figure 1:**
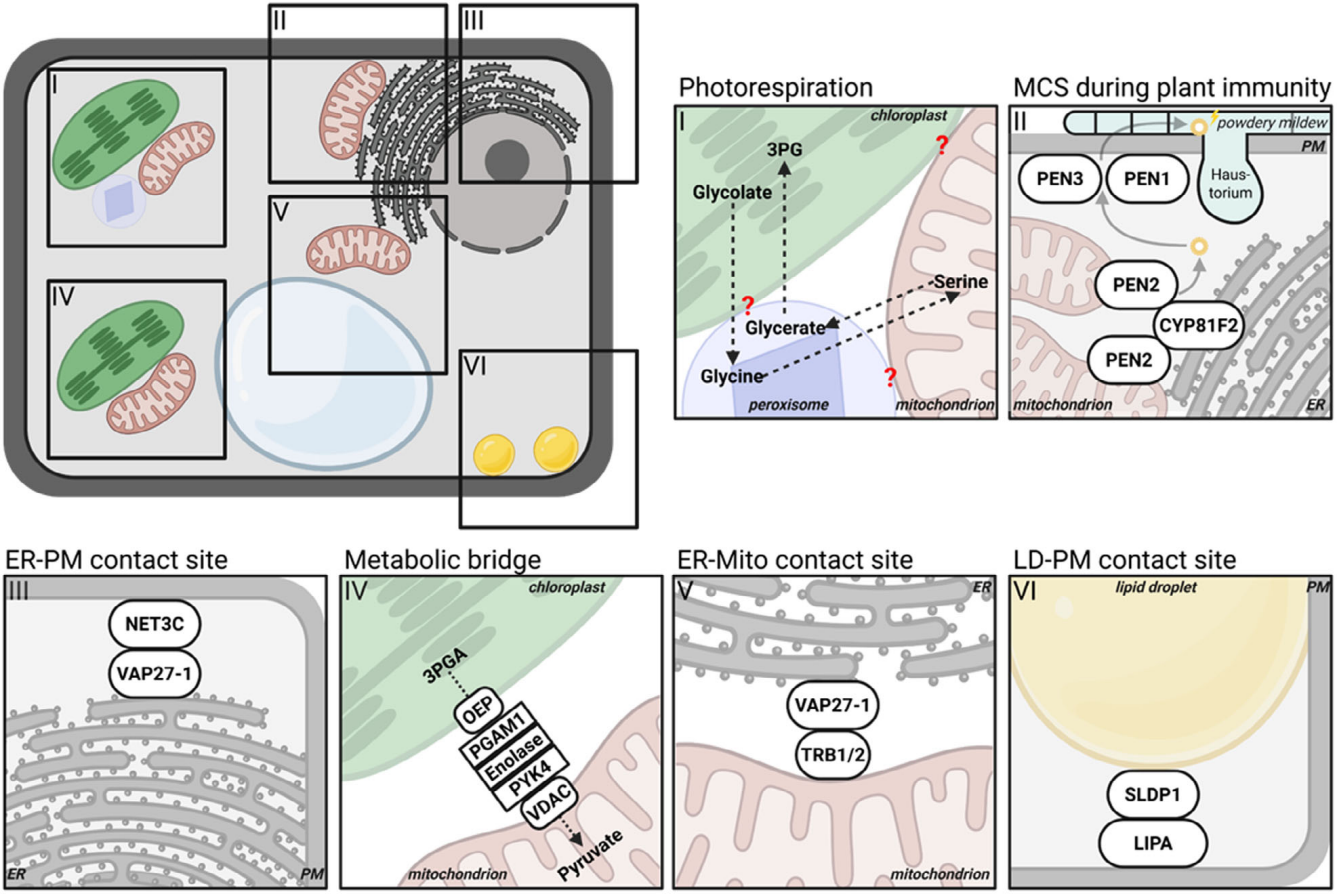
Examples for specific organelle positioning with likely metabolic significance. (I) Photorespiration involves the exchange of metabolites between chloroplasts, mitochondria, and peroxisomes. (II) Endoplasmic reticulum (ER) and mitochondria are positioned near fungal invasion sites, where CYP81F2 synthesizes modified indole glucosinolates, PEN2 converts them into antimicrobial compounds, and PEN1 together with PEN3 directs these defense compounds to the infection site (based on Fuchs et al., 2016). (III) The ER can be anchored to the plasma membrane (PM) via the NET3C‐VAP27‐1 complex (based on Wang et al., 2023). (IV) Metabolic bridges between chloroplasts and mitochondria facilitate efficient metabolite transport (based on Zhang et al., 2020). (V) The VAP27‐1‐TRB1/2 complex forms contact sites between ER and mitochondria (based on Li et al., 2022). (VI) SLDP1 and LIPA establish contact sites between lipid droplets and the plasma membrane (based on Krawczyk et al., 2022). Created in BioRender. Schwarzlander (2026). https://BioRender.com/d2w0yd4.

Plants have evolved elaborate acclimation strategies when challenged with adverse environmental conditions (Kleine et al., 2021; Schwenkert et al., 2022). Jointly, those mechanisms replace the need to physically change location. The ability of plants to acclimate involves all levels of their organization. Environment‐triggered changes determine plant developmental plasticity as a key distinction from most animals that typically have a fixed body plan (Jiang et al., 2025; Schneider et al., 2026). This plasticity includes tropic and nastic movements that are implemented through changes in plant growth, typically cell elongation (Harmer & Brooks, 2018). At the cellular level, those changes are mediated by intracellular signaling and the adjustment of membrane transport, gene expression, and metabolic status. The cells themselves, however, remain largely fixed in their position relative to their neighbors through the rigidity of apoplastic cell wall structures. By contrast, their intracellular composition is highly dynamic and defined by intense cytoplasmic movement as powered by cytoskeletal transport, as mainly mediated by myosin (Peremyslov et al., 2015; Kurth et al., 2017). The relative positioning of the different organelles toward another is far from chaotic, however. Defined arrangements are strictly required for cell division and normal development (Sheahan et al., 2004; Koç & De Storme, 2022), but beyond this, specific organelle positioning is involved in the responses to biotic and abiotic stresses (Wada & Suetsugu, 2004, Fuchs et al., 2016). The functional significance of the movement of nuclei and chloroplasts has received major attention, for example, in optimization of photosynthesis, while the movement of mitochondria, peroxisomes and endoplasmic reticulum tubules, Golgi and other endomembrane vesicles as well as lipid droplets has mainly been investigated at the phenomenological level (Evans & Hawes, 2010; Logan, 2010; Midorikawa et al., 2022; Oikawa et al., 2003; White et al., 2020) (Figure 1). The emerging picture suggests that the intracellular positioning of individual organelles is tightly controlled in response to developmental and environmental stimuli. Nevertheless, the underlying mechanisms and the physiological significance of their positioning remain unclear in many aspects. In this perspective article, we review selected examples of organelle positioning that likely contribute to the regulation of cellular metabolism. We then make use of the recently described example of a physical link between mitochondria and chloroplasts by three glycolytic enzymes (Zhang et al., 2020) to highlight conceptual approaches required to further scrutinize and develop the conceptual framework of how the relative spatial arrangement of organelles toward another may act as a regulatory principle of metabolism and stress responses.

Based on their significance as hubs of cellular metabolism we set a focus on our current understanding of chloroplast and mitochondrial positioning to explore the role of organelle positioning in the regulation of (stress) metabolism. The principles and hypotheses that we derive may be generalized, however, and deserve dedicated testing also for the positioning of other organelles.

## INTRACELLULAR POSITIONING OF CHLOROPLASTS

A major driver for chloroplast movement is light (Kihara et al., 2020; Midorikawa et al., 2022; Wada et al., 2003). To avoid photodamage and to maximize light capture, chloroplasts move toward low light and away from high light. Intracellular chloroplast movement utilizes actin filaments, which interact with the chloroplast outer membrane protein CHUP1 (Kong et al., 2024; Von Braun & Schleiff, 2008; Wilson & Ruban, 2020). Genetic disruption of chloroplast positioning was found to impair metabolic and photosynthetic acclimation in response to cold (Kitashova et al., 2021). Light‐induced chloroplast movement is controlled by the blue‐light receptors PHOT1 and PHOT2 (Sakai et al., 2001; Wilson & Ruban, 2020). C4 plants show cell‐type‐specific positioning of their chloroplasts. Bundle sheath cell chloroplasts are permanently and polarly anchored centripetally (close to the vascular bundle) or centrifugally (close to the mesophyll) positioned depending on species. In contrast, chloroplasts in mesophyll cells are more dynamic and can relocate, to aggregate toward the bundle sheath under stress conditions (Kato et al., 2022). Together, these observations highlight that chloroplast positioning is tightly linked to the optimization and protection of photosynthetic metabolism.

## INTRACELLULAR POSITIONING OF MITOCHONDRIA

Mitochondria of seed plants are particularly dynamic, undergoing continuous fission and fusion and rapidly changing their cellular distribution (Logan, 2006). While in animal cells, abnormal mitochondrial distribution results in severe developmental impairments and neurodegenerative diseases (Wang et al., 2020), the importance of mitochondrial distribution for plant growth and development remains insufficiently understood. Logan et al. (2003) identified several mutations that impaired mitochondrial morphology and dynamics. One of these, *friendly mitochondria* (*fmt*), was mapped to a homolog of the CLUSTERED MITOCHONDRIA (CLU) superfamily, and resulted in impaired growth (El Zawily et al., 2014). More recently, FMT was found to be involved in translation at the mitochondrial surface and in mitophagy of damaged mitochondria (Hemono et al., 2022; Kacprzak & Van Aken, 2023; Ma et al., 2021). However, whether there is a causal relation between altered mitochondrial positioning in *fmt*—or any of the other known mutants affecting mitochondrial positioning (Table 1)—and growth defects remains difficult to delineate, since impaired translation and mitophagy are likely to have pleiotropic effects. Thus, despite their pronounced intracellular mobility, the functional significance of mitochondrial positioning for metabolic regulation in plants remains largely unresolved.

**Table 1:** Non‐comprehensive list of selected Arabidopsis mutant lines with changed organelle positioning

Organelle	Mutant	Involved in	Mutant description	References
Chloroplasts	*phot1 & phot2*	Blue‐light sensing & light movement	Changed positioning responses to light (accumulation & avoidance movement)	Sakai et al. (2001) Jarillo et al. (2001) Kagawa et al. (2001)
Chloroplasts	*phyA & phyB*	Red/far‐red light movement	Impaired contribution to blue light‐dependent movements by modulating the transition between the high‐ & low‐light responses meditated by *phot1* & *phot2*	DeBlasio et al. (2003)
Chloroplasts	*chup1*	Anchoring & Actin‐based movement	Fail to undergo normal light‐induced relocation (accumulate in basal region of mesophyll cells instead of proper periclinal/anticlinal distribution)	Kasahara et al. (2002) Oikawa et al. (2003)
Chloroplasts	*kac1 & kac2*	Attachment to cp‐Actin filaments	Lacking photorelocation movement; detachment from the plasma membrane	Suetsugu et al. (2010)
Chloroplasts	*web1 & pmi2*	Regulation of light avoidance response	Impairment of blue light‐induced chloroplast‐Actin filament reorganization, lowering speed	Kodama et al. (2010) Luesse et al. (2006)
Chloroplasts	*jac1*	Accumulation response	Impaired accumulation response under weak blue light; defective in chloroplast movement in darkness	Suetsugu et al. (2005)
Chloroplasts	*pmi1*	Movement signaling	Changed low‐ & high‐light‐dependent movement	DeBlasio et al. (2005)
ER	*net3c & vap27*	ER‐plasma membrane contact sites	Impairment of ER & PM contact sites	Wang et al. (2014) Wang et al. (2023)
ER	*syt1*	ER membrane stability & ER‐plasma membrane tethering	Changed ER stability & regulation of vesicle trafficking by controlling the extent of ER‐PM contact sites	Siao et al. (2016)
ER	*ermo1 & ermo2*	Morphology organization	Increased number of ER‐derived spherical bodies throughout *ermo1* cells, large aggregates in *ermo2*	Nakano et al. (2009)
ER & mitochondria	*trb1, trb2, & vap27*	ER‐mitochondria contact sites	Impaired mitophagy & ER‐interaction of mitochondria	Li et al. (2022)
ER & Golgi	*rhd3*	ER tubule fusion & ER‐Golgi trafficking	Disorganization of the ER & Golgi stacks	Zheng et al. (2004)
ER & Golgi	*mag4*	Golgi‐to‐ER transport	Impaired tether formation	Takahashi et al. (2010)
ER & Golgi	*casp*	ER‐Golgi secretory pathway organization	Golgi body speed & displacement were significantly reduced; ER‐Golgi connection was more easily disrupted	Osterrieder et al. (2017)
Golgi	*crooked*	Distribution & morphology	Structure of Actin filaments is altered; abnormal accumulation of some Golgi stacks at certain regions in trichomes	Mathur et al. (2003)
Golgi	*kam1*	Motility & Actin organization	Golgi‐Aggregates (Actin filaments interact with Golgi stacks via KAM1 to maintain the proper organization of endomembranes)	Tamura et al. (2005)
Golgi	*kinesin‐13a*	Positioning & microtubule interaction	Aggregation/clustering of Golgi stacks in trichomes & other epidermal cells	Lu et al. (2005)
Golgi	*gnom*	Vesicle trafficking & Golgi‐mediated recycling	TGN/EE compartments aggregate or become enlarged	Naramoto et al. (2014)
Lipid droplets	*sldp1, sldp2, & lipa*	Anchoring to ER	Aberrant clustering in seedlings	Krawczyk et al. (2022)
Lipid droplets	*myo11c1‐1 & myo11c2‐1*	Organelle movement	Trafficking slowed down in pollen tubes	Yang et al. (2023)
Mitochondria, Golgi & peroxisomes	*xi‐k, xi‐1, xi‐2, & xi‐i*	Organelle transport along Actin filaments	Reduced organelle motility, changed distribution	Peremyslov et al. (2010) Peremyslov et al. (2008)
Mitochondria	*msh1*	Genome maintenance	Less evenly spread mitochondria, networks of inter‐mitochondrial encounters become more connected; MSH1 is also present in plastids, which were not assessed for positioning in the mutant, however	Chustecki et al. (2022)
Mitochondria	*fmt*	Dynamics & distribution	Clustered mitochondria	El Zawily et al. (2014)
Mitochondria	*nmt*	Morphology & trafficking	Tubular mitochondria, these tubules frequently form interconnecting networks	Logan et al. (2003)
Mitochondria	*pmf1 & pmf2*	Fusion & morphology	Reduced mitochondrial volume & impaired fusion during hypoxic stress	Kenneally et al. (2026)
Mitochondria	*miro1*	Movement & positioning	Pollen exhibits abnormally enlarged or tube‐like mitochondrial morphology, disruption of continuous streaming in the growing pollen tube	Yamaoka and Leaver (2008) Lu et al. (2026)
Mitochondria	*miro2 ssnn* (GTPase inactive)	Movement & positioning	Increased mitochondrial number, decreased mitochondrial size, increased fission, weakened mitochondria‐ER‐connection	White et al. (2020)
Mitochondria	*phot1 & phot2*	Light‐dependent positioning	Involved in the early acceleration of mitochondrial movement (at blue light)	Islam et al. (2020)
Mitochondria & peroxisomes	*fis1a & fis1b*	Organelle fission/division	Decreased mitochondrial & peroxisomal numbers, increase in size of individual peroxisomes & mitochondria; FIS1A is also present at plastids, which were not assessed for positioning in the mutant, however	Zhang and Hu (2008) Scott et al. (2006) Ruberti et al. (2014)
Mitochondria & peroxisomes	*drp3a & drp3b*	Organelle fission/division	Fusion, aggregation, tubulation of mitochondria, larger spherical mitochondria, decreased number Elongated peroxisomes	Arimura and Tsutsumi (2002) Logan et al. (2004) Zhang and Hu (2009)
Peroxisomes	*peup1, peup2, & peup4*	Positioning & inheritance	Aggregated & increased number	Shibata et al. (2013)
Peroxisomes	*apem1, apem3, & apem10*	Biogenesis & protein import	Elongated, enlarged & decreased number	Mano et al. (2004) Mano et al. (2011) Goto‐Yamada et al. (2014)
Peroxisomes	*pex11*	Proliferation & division	Reduced number	Orth et al. (2007)

## POSITIONING OF CHLOROPLASTS, MITOCHONDRIA AND PEROXISOMES IN PHOTORESPIRATORY METABOLISM

Under photorespiratory conditions, chloroplasts, mitochondria, and peroxisomes associate, aiding the efficient flux of photorespiratory metabolites (Koenig et al., 2023). CO_2_ released during photorespiration is thought to be lost, rendering C3 photosynthesis inefficient, especially under conditions of elevated, atmospheric temperature (Fernie & Bauwe, 2020; Segura Broncano et al., 2023). Several metabolic bypasses to endogenous pathways have been engineered into transgenic plants, giving rise to improved biomass production (Eisenhut et al., 2017; Meacham‐Hensold et al., 2026). While the reported biomass increases in several of these transgenic plants are remarkable, the view of a single function of photorespiration has been questioned (Eisenhut et al., 2017; Fernie & Bauwe, 2020; Peterhansel et al., 2013). Instead, photorespiration is likely to also contribute to nitrogen and sulfur assimilation, C1 metabolism, and mitochondrial redox balance (Fernie et al., 2013; Rosa‐Téllez et al., 2024; Sweetlove et al., 2006). It is important to note that considerable gaps in our understanding of photorespiration remain. For example, it has recently been demonstrated that growth under fluctuating light buffers plant growth and metabolism against photorespiratory perturbations (von Bismarck et al., 2023), a finding that contradicts previous claims that photorespiration was essential for the adaptation to such conditions (Huang et al., 2015). This renders interpretations of experiments on photorespiration somewhat difficult. Considering this degree of uncertainty, the positioning of other organelles relative to the chloroplast may play a role in other aspects of photosynthetic metabolism, that is, beyond photorespiration (Midorikawa et al., 2022). However, in these instances, the exact reasons and mechanisms have yet to be explored. An interesting example for metabolic collaboration between mitochondria and peroxisomes was recently reported in mammalian cells, where their close association is critical for mitochondrial glutathione redox maintenance through the detoxification of mitochondria‐derived ROS in peroxisomes (DiGiovanni et al., 2025). Since in plant cells the chloroplast is an additional source of high quantities of H_2_O_2_, which strongly depends on the illumination regime, and since chloroplast‐derived H_2_O_2_ was found to also reach other compartments (Ugalde et al., 2021), it is tempting to speculate about an analogous role for the juxtaposed positioning of peroxisomes, mitochondria and chloroplasts in plant cells. In line with such a hypothesis, absence of peroxisomal Catalase 2 (CAT2) or stromal Ascorbate Peroxidase were found to lead to oxidation in the mitochondrial matrix, but not the cytosol (Nietzel et al., 2019). Since this observation was made in root cells, it deserves follow up in photosynthetically active tissues under different illumination regimes.

## GLYCOLYTIC ENZYMES COORDINATE METABOLISM AND POSITIONING OF CHLOROPLASTS AND MITOCHONDRIA

Different glycolytic enzymes can form transient protein assemblies, which have been observed across the kingdoms of life (Araiza‐Olivera et al., 2013; Samuel Russell et al., 2025; Sweetlove & Fernie, 2018). In plants, all glycolytic enzymes can physically associate with mitochondria and sustain respiratory flux with glucose as substrate (Giegé et al., 2003). The association is dynamic and dependent on the respiratory demand. The addition of respiratory uncouplers decreases the association, while enhanced substrate supply increases it (Giegé et al., 2003; Graham et al., 2007; Zhang et al., 2023). However, while a picture is starting to emerge for the interactome of the tricarboxylic acid (TCA) cycle (Jung & Mack, 2018; Omini et al., 2021; Senkler et al., 2017; Zhang et al., 2017, 2018), the significance of glycolytic assemblies is less well understood. A recent study found that Phosphoglycerate Mutase and Enolase form a substrate‐channel, which is part of a larger protein complex, including Pyruvate Kinase (Zhang et al., 2020). Kinetic experiments suggest that this association allows for more efficient conversion of the glycolytic intermediates. Disruption of this complex diminished the association of chloroplasts and mitochondria, revealing a moonlighting role for these enzymes in mediating the juxtaposed positioning of these organelles (Zhang et al., 2020). The data indicated that Phosphoglycerate Mutase and Enolase form a metabolon bound to the outside of the mitochondria via association with Pyruvate Kinase and the Voltage‐Dependent Anion Channel (VDAC). Also, the previously described full plant glycolytic metabolon (Giegé et al., 2003; Graham et al., 2007) was recovered, confirming interactions between glycolytic enzymes and cytoskeletal components (Araiza‐Olivera et al., 2013; Garagounis et al., 2017) and providing evidence of a putative association between the phosphoglycerate mutase–enolase–pyruvate kinase complex and the Triose Phosphate Transporter (TPT) and Outer Envelope Protein 21 (OEP21). In such a scenario, OEP21 is a candidate to physically link TPT as an inner envelope protein, with the glycolytic enzymes on the cytosolic surface of the outer envelope. The combination of several orthogonal techniques strongly suggested a moonlighting role of lower glycolysis in promoting the efficient coordination of the major energy transformation systems of the plant cell. Elucidating the critical amino acid residues that are involved in the formation of the complex will be essential to develop the tools to assess the cellular and organismal significance of the dynamic association between glycolytic proteins and membrane proteins of both the mitochondrial and chloroplast membrane systems. Those tools should then be used to dissect the dynamics and the physiological purpose of the ‘enzyme bridge’.

## METABOLIC NANOENVIRONMENTS AND COMPARTMENTALIZED ENERGY AND REDOX DYNAMICS

Precise organelle positioning as a metabolic control principle is part of a more general concept of physically structured metabolism for dynamic regulation (Sweetlove & Fernie, 2013). In compact cellular geometries, like those of most plant cells, diffusion across the cytosol is expected to be efficient (Sweetlove & Fernie, 2013), and theoretical considerations have been arguing against any major gradients, for instance of ATP, in the eukaryotic cytosol; at least under standard conditions (Kumar & Johnston, 2026). Nonetheless, gradients set by source and sink activities within a cell compartment have been detected for simple ions such as Ca^2+^ and H^+^, for example, in the cytosol of tip growing cells, like pollen tubes or root hairs, and even within individual mitochondrial cristae (Li et al., 2021; Rieger et al., 2014). They also remain plausible for ATP under specific conditions for example, of high source–sink flux (Kumar & Johnston, 2026).

Recently, our current assumptions that diffusion provides efficient homogenization within cells and organelles, including biomolecular condensates, have been challenged (Ausserwöger et al., 2026; Lee et al., 2025). In plant cells, limitations to diffusion may be particularly pronounced due to the dominance of chloroplasts and the vacuole, which constrain the cytosolic space and may favor the emergence of localized metabolic nanoenvironments. Liquid–liquid phase separation may further contribute to structuring the cytosol by forming transient biomolecular condensates that can locally concentrate enzymes and modulate metabolic fluxes (Emenecker et al., 2020; Solis‐Miranda et al., 2023; Sweetlove & Fernie, 2018).

Those findings make it seem plausible that metabolic gradients and nanoenvironments in the plant cytosol may indeed result from the ambient physical and physiological constraints. To test whether such a level of metabolic structure really exists in cells, systematic empirical assessment through the establishment of dedicated approaches, including the deployment of genetically encoded nanosensors (Niemeier et al., 2026), will be essential. Both the existence of metabolite gradients and nanodomains, and potential physiological roles in metabolic regulation will need to be critically assessed and specific models will need to be established for proof‐of‐concept.

**Figure 2:**
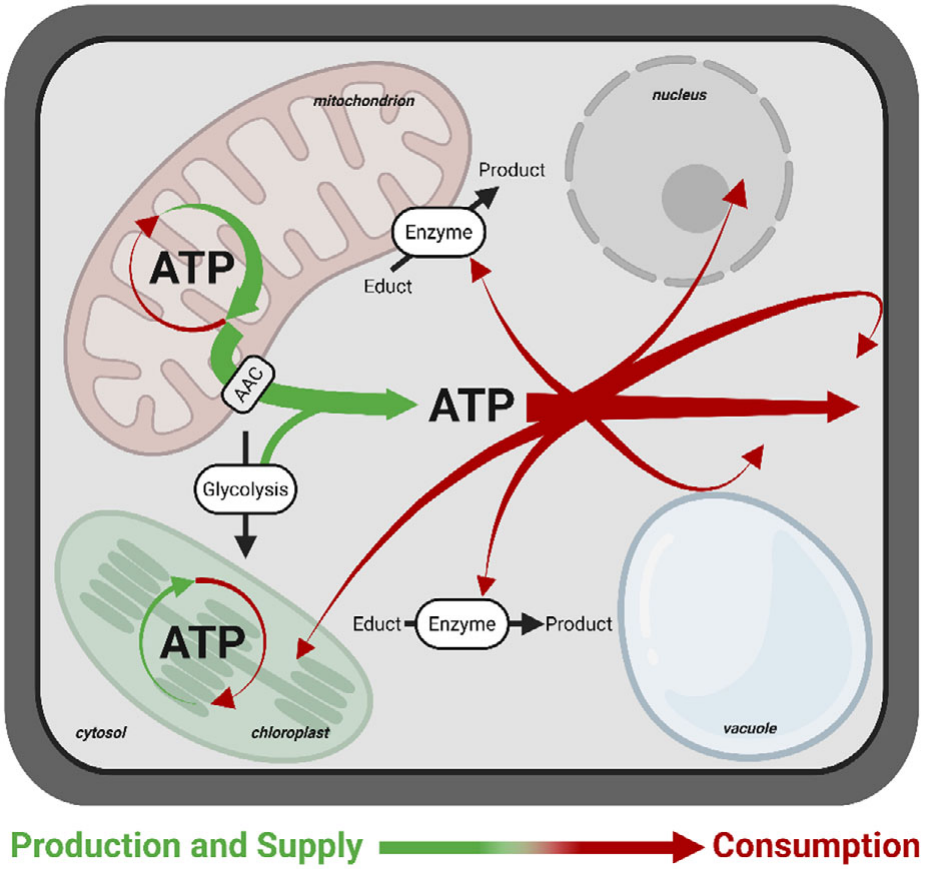
Metabolite fluxes are constrained by the spatial organization of metabolism, as illustrated by ATP production, transport, and consumption. Hotspots of ATP production are located in mitochondria, chloroplasts, and the cytosol. ATP generated in mitochondria can be exported to fuel cytosolic or other organelles' metabolism, whereas chloroplasts retain their ATP, where it is also consumed. Note that import of ATP from the cytosol into the stroma occurs predominantly under specific circumstances, such as at night, in developing chloroplasts and in heterotrophic tissues (Reinhold et al., 2007; Reiser et al., 2004). Major cellular ATP sinks are the ATPases at the plasma membrane and the endomembrane system, as well as cytosolic and nuclear processes in cytoskeletal dynamics, biosynthesis, and gene expression. Created in BioRender. Schwarzlander (2026). https://BioRender.com/d2w0yd4.

Specifically, spatial heterogeneity is expected to manifest in local differences in energy status and the potential of redox‐active compounds in a given cell compartment. Mitochondria and chloroplasts are both hubs of ATP generation in photosynthesizing plant cells (Carrari et al., 2003; Nunes‐Nesi et al., 2007; De Col et al., 2017; Voon et al., 2018; Igamberdiev & Bykova, 2023; Yang et al., 2024) (Figure 2). While mitochondrial ATP is efficiently exported to the cytosol, stromal ATP is not at any physiologically meaningful rate and is rather consumed directly within the stroma (Shameer et al., 2019; Vera‐Vives et al., 2024). Under specific conditions, such as during the night, ATP even needs to be imported from the cytosolic ATP pool into the chloroplast stroma through the nucleotide transporters of the inner envelope (Reinhold et al., 2007; Reiser et al., 2004). Instead of the direct export of ATP molecules, adenylate charge is exported to the cytosol via metabolic shuttle systems, such as the triose phosphate shuttle (Fischer & Weber, 2017; Sharkey, 2024). In the cytosol, glycolysis can also contribute to gross ATP production (Mira et al., 2023). Considering those specific constraints, the *in vivo* impact of organelle positioning on cytosolic ATP dynamics deserves investigation. The same is true for other central metabolic cofactors, such as NAD and NADP, which act as key determinants of subcellular redox metabolism, including glycolytic flux and detoxification (Feitosa‐Araujo et al., 2022; Smith et al., 2021). In addition to the triose phosphate shuttle, the reducing power from the chloroplast NADP pool is efficiently linked to the NAD pool of the cytosol via the malate/oxaloacetate shuttle (Taniguchi et al., 2002; Zheng et al., 2025). These shuttles connect prominent subcellular redox pools and integrate metabolism across compartments (Scheibe et al., 2005). Subcellular fractionation delivered elegant insight into the compartmentation of metabolites and cofactors (Farré et al., 2001; Heber & Santarius, 1965; Tiessen et al., 2002; Tohge et al., 2011). Yet, the insight into metabolic dynamics has remained limited. Because information about organelle metabolism is lost by disruptive approaches, future experimental efforts are likely to require the optimization of suitable *in vivo* approaches.

## TOWARD THE SYSTEMATIC ASSESSMENT OF THE PHYSIOLOGICAL ROLES OF ORGANELLE POSITIONING

The above examples highlight the concept that plant cells control the positioning of their organelles to coordinate metabolic pathways among them. While anatomical studies, typically using microscopy techniques, allow correlation of organelle distribution patterns with metabolic performance, on their own, they typically cannot establish cause‐and‐effect relationships. Mutants that show aberrant organelle positioning, as well as genetic screens to isolate novel mutants (Logan et al., 2003), provide a promising starting point to establish such causal relationships (Table 1).

To directly assess the functional significance of organelle positioning, experimental strategies are required that allow controlled and reversible manipulation of organelle spatial reorganization. Interventional repositioning of organelles at the single‐cell level has been elegantly achieved using optical tweezers (Gao et al., 2016), but their applicability is limited to highly localized measurements. For broader physiological insight, genetic and inducible systems that modulate organelle proximity across tissues are likely to be more powerful (Table 2). Complementary approaches to monitor the metabolic consequences of such perturbations, including genetically encoded biosensors and quantitative imaging, will be essential (Table 3). In parallel with live‐cell imaging, future investigations would also benefit from complementary biochemical and ultrastructural approaches. Crosslinking mass spectrometry (Liu et al., 2018) could help define transient protein assemblies and interaction hubs at organelle contact sites, while electron microscopy and tomography (Liang et al., 2018; Wang et al., 2019) may allow direct visualization of the spatial arrangement and physical proximity of organelles *in vivo*. Together, these approaches establish a framework to experimentally dissect how organelle positioning shapes cellular metabolism.

**Table 2:** Tools to manipulate organelle positioning & its metabolic significance

Approach	Principle	Metabolic significance
Optical tweezers	Laser‐based trapping allows physical repositioning of (single) organelles in living cells	Enables testing how spatial positioning of organelles influences metabolic interactions & energy distribution within the cell
Artificial organelle tethers	*Constitutive synthetic linkers*: Engineered proteins with two distinct organelle‐targeting domains that permanently bridge two organelles	Allow precise control over the proximity of organelles, enabling the study of how spatial organization affects metabolism. Constitutive tethers reveal the effects of stable contacts, inducible tethers allow temporal manipulation to study dynamic processes, & contact‐FP systems provide both visualization & modulation of contacts
	*Chemically inducible systems*: Two engineered proteins are expressed on separate organelles. Addition of a specific molecule (e.g., rapamycin) triggers tether formation	
	*Contact fluorescent protein sensors (contact FPs/split‐FP systems)*: Split fluorescent protein fragments are targeted to two different organelle membranes. Only when the organelles come within a critical proximity do the fragments reassemble & fluoresce.	
Cytoskeleton perturbation	Chemical or genetic disruption of Actin filaments or microtubules (e.g., by Latrunculin B)	Reveals how organelle movement contributes to metabolic homeostasis & intracellular metabolite distribution
Motor protein manipulation	Genetic or chemical (e.g., by pentabromopseudilin) modification of myosin motors controlling organelle transport	

**Table 3:** Tools to monitor organelle positioning & its metabolic significance

Approach	Principle	Metabolic significance
Genetically encoded fluorescent biosensors	Fluorescent protein‐based biosensors enable real‐time monitoring in living cells. Targeting sequences allow measurements in specific (single) compartments or at organelle membranes; for example, ATP, metabolite (for glucose or pyruvate), NAD(P)H/NAD(P)^+^ & pH sensors	Enable spatially resolved measurements of metabolic states at organelle membranes or within specific compartments, revealing local metabolic environments
Biosensor readout analysis	Quantification of metabolite levels by calculating fluorescence ratios (e.g., Fricker, 2016)	Allows robust comparison of metabolic states across cells, time points & subcellular regions, enabling quantitative analysis of local metabolic changes
Particle tracking & trajectory analysis	Computational tracking of organelle trajectories to quantify velocity, displacement & interaction frequency (e.g., Arivis Vision4D, TrackMate)	Enables quantitative analysis of organelle dynamics
Metabolic & biochemical analysis	*Targeted metabolomics*: Quantification of metabolites in cellular extracts	Provides accurate profiles of central metabolic pools & energy status, allowing correlation of organelle positioning or contact site perturbations with changes in metabolic state
	*Enzyme activity assays*: Biochemical measurement of the catalytic rates of key metabolic enzymes	Connects changes in organelle morphology/contacts to functional metabolic capacity
	*Proximity labelling (e.g., TurboID)*: Biotin ligase fused to organelle/contact‐site proteins biotinylates proximal proteins which can be identified by mass spectrometry	Enables identification of protein communities at membranes & contact sites under different metabolic states

## SYNTHETIC ORGANELLE POSITIONING AND INTERACTION

A key requirement to test the functional role of organelle positioning is the ability to manipulate organelle proximity in a controlled manner. An elegant and innovative manner to covalently link proteins and create artificial multiprotein assemblies is the use of molecular superglue systems based on engineered Ig‐like domains (Reddington & Howarth, 2015; Veggiani et al., 2014; Zakeri & Howarth, 2010). A particularly efficient, well‐ characterized and therefore widely used post‐translational protein coupling system, consisting of the SpyCatcher and SpyTag components, can be used for stable, rapid, irreversible, and specific linkage of proteins (Gilbert et al., 2017; Reddington & Howarth, 2015). In mammals, this system has been used for stabilizing enzymes and vaccine optimization, as well as in the defined construction of large, multi‐component architectures. The composite connections were, furthermore, demonstrated to be highly stable under a wide range of conditions, including heat, pH, detergents, and mechanical forces. A two‐component system was developed that allows to synthetically manipulate the association between organelles by mediating the specific covalent linkage of two co‐expressed peptides *in planta* (Lang et al., 2020). So far, the system has been targeted to the outer envelopes/membranes of chloroplasts, mitochondria, tonoplast, and nuclei (Lang et al., 2020). Tags and catchers are generated by two protein domains that can autocatalytically form intramolecular isopeptide bonds under physiological conditions. Starting from the first tag/catcher system (Zakeri & Howarth, 2010), additional tag/catcher pairs have been developed which allow multiplexing of sequence‐specific intermolecular isopeptide bonding (Lang et al., 2020). SpyCatcher and SpyTag can be used to test organelle repositioning *in planta* by co‐expressing the components targeted to either the outer chloroplast envelope or the outer mitochondrial membrane. The coexpression of both tags is anticipated to result in the enhanced association of mitochondria and chloroplasts, which would not take place if a single tag were expressed alone. A range of alternative approaches with different properties is available and deserves empirical comparison in plant (cell) systems (Table 2), such as optimization of expression strength and timing of the analysis after induction. Such a strategy to synthetically reorganize organelle interactions would allow dissecting the significance of their positioning for metabolic regulation and stress responses.

## *IN PLANTA* BIOSENSING OF MgATP^2^ ^−^ AND NAD(P)H/NAD(P)^+^

Strategies to manipulate organelle positioning require complementary approaches to assess the metabolic consequences *in vivo*. To investigate how organelle positioning shapes metabolic states, spatially resolved readouts are required. The fluorescent protein probe ATeam1.03‐nD/nA (Kotera et al., 2010) was used to develop MgATP^2−^ biosensing in living plants (De Col et al., 2017). *In vitro* assessment revealed specificity for the MgATP^2−^ complex (the biologically most relevant form of ATP), as well as pH stability. Targeting this probe to the cytosol and the chloroplast stroma allowed monitoring ATP changes live at illumination and revealed a steep gradient in MgATP^2−^ concentration between the cytosol and the chloroplast stroma in leaf mesophyll (Elsässer et al., 2020; Voon et al., 2018). While mitochondrial targeting remains problematic in plants, changes in mitochondrial ATP production could be monitored thanks to the efficient ATP export from the mitochondria to the cytosol (De Col et al., 2017). Under the assumption of a stable total adenylate pool, ATP concentration changes represent changes in adenylate charge. Another sensor that responds to the ATP:ADP ratio has been tested, but its use is limited in plants due to a pronounced pH sensitivity (Tantama et al., 2013).

Recently NADH/NAD^+^ biosensing *in planta* based on the Peredox‐mCherry biosensor was optimized (Hung et al., 2011; Steinbeck et al., 2020). Light exposure triggered a rapid reduction of the cytosolic NAD pool, indicating reductant export from the plastid. Similar observations were independently linked to photorespiratory reductant reallocation (Lim et al., 2020). Suppression of mitochondrial respiration through hypoxia or electron transport inhibitors also led to a fast reduction of the cytosolic NAD^+^ pool (Wagner et al., 2019). Moreover, manipulating glycolytic flux and the function of the mitochondria or the chloroplasts had a synergistic impact on the cytosolic NAD redox status (Steinbeck et al., 2020).

To explore the NADP redox dynamics *in planta*, a new set of pH‐stable and highly specific biosensors, the NAPstars, was recently developed (Scherschel et al., 2024). This innovation has filled a critical gap left by biosensors for NADPH concentration that were previously adopted and optimized for the use in plants (Lim et al., 2020, 2024); recently reviewed in Niemeier et al. (2026). The NAPstar biosensors were used to demonstrate how the NADP pool delivers reductant for cytosolic H_2_O_2_ detoxification and to highlight the interconnectivity of the chloroplast and the cytosol by monitoring the export of reducing power from the stromal NADP pool to the cytosolic NAD pool under illumination. Because actinic illumination interferes with fluorescent biosensing, a custom illumination setup for live imaging by confocal microscopy by either using red light or by synchronizing on‐stage sample illumination with laser scanning cycles was developed (Degen et al., 2025; Kuang et al., 2025; Uflewski et al., 2024; Vera‐Vives et al., 2024; Zheng et al., 2025). Prior observations provide proof‐of‐concept that metabolic signatures can be resolved by biosensing (De Col et al., 2017; Lim et al., 2020, 2024; Scherschel et al., 2024; Steinbeck et al., 2020; Vera‐Vives et al., 2024; Voon et al., 2018), which is an important prerequisite for the study of the role of organelle positioning in setting metabolic nanoenvironments.

**Figure 3:**
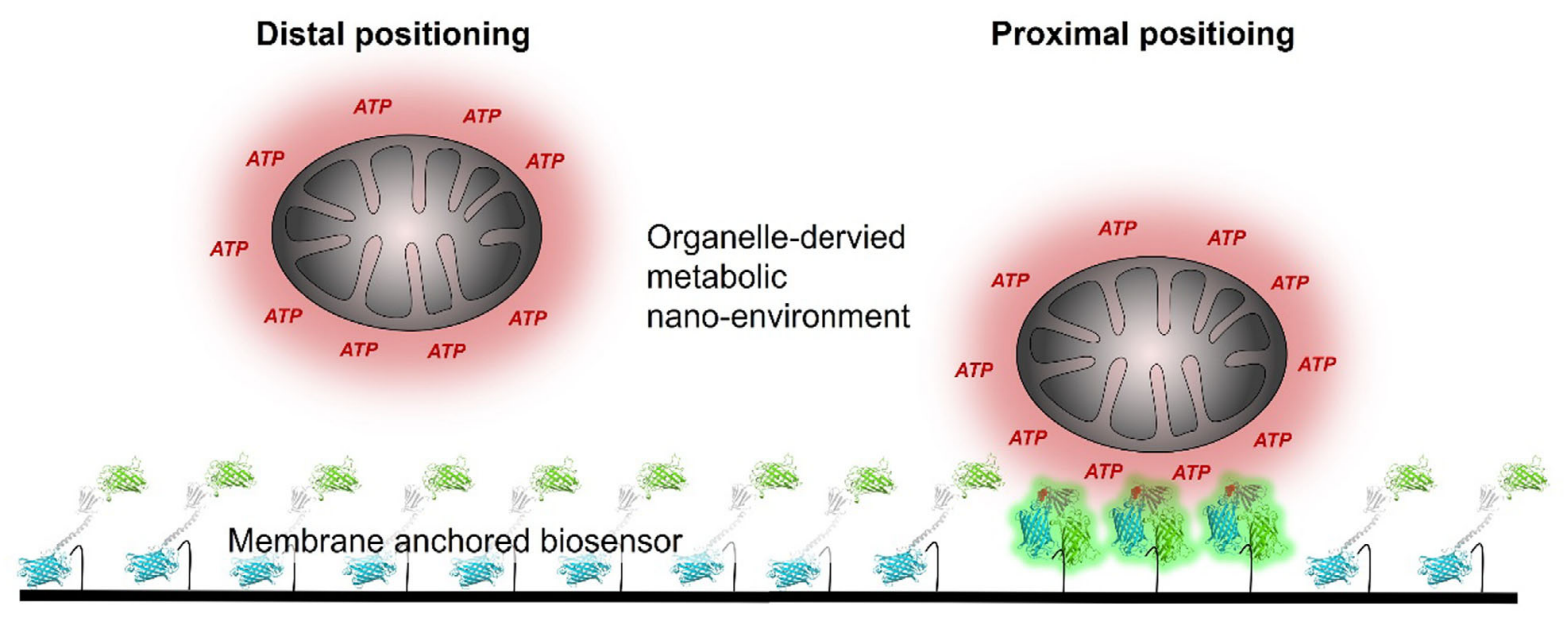
Concept of metabolic nanoenvironment assessment by membrane anchored biosensors. Example illustrates the ATP‐biosensor ATeam1.03‐nD/nA to be fused to the cytosolic face of a defined membrane system to detect ATP nano‐environments around cell organelles such as mitochondria depending in their proximity.

Not only the positioning, but also the surrounding nanoenvironment of an organelle may affect its energy state and its redox couples. In order to test the hypothesis that organelles shape a heterogenic metabolic landscape within the cytosol, the ATeam1.03‐nA/nD, Peredox‐mCherry, and NAPstar could be immobilized in the outer mitochondrial membrane, the chloroplast envelope, the tonoplast, the nuclear envelope, and the plasma membrane facing the cytosol (Figure 3). The membrane localization would constrain sensor diffusion by one dimension to discriminate local nanoenvironments of ATP and NAD(P) redox status directly at the membrane surface. The plasma membrane and the tonoplast host H^+^‐ATPases as major ATP sinks, and their activity can vary strongly depending on conditions. In analogy, major consumption of NADPH is expected in close proximity to the NADPH‐dependent Respiratory Burst Oxidase Homologous upon their activation to generate apoplastic ROS bursts (Torres et al., 1998).

## STABILIZING AND MONITORING THE GLYCOLYTIC METABOLON BETWEEN MITOCHONDRIA AND CHLOROPLASTS

**Figure 4:**
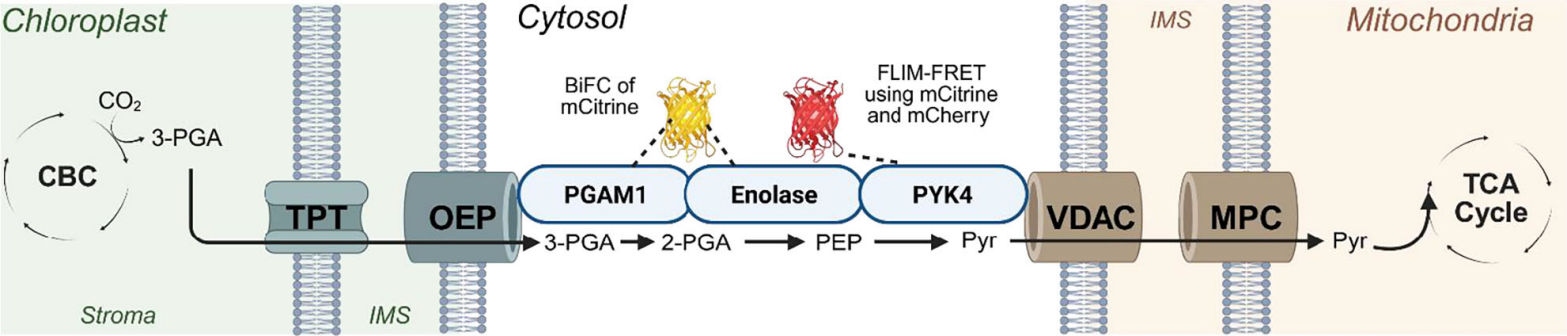
Engineering of a semi‐constitutive glycolytic enzyme complex between mitochondria and chloroplast *in planta* and monitoring its response to altered metabolic status. The mCitrineNE‐enolase and phosphoglycerate mutase (PGAM1)‐mCitrineCE are linked irreversibly through Bimolecular Fluorescence Complementation (BiFC) of mCitrine. Enolase/iPGAM1‐mCitrine dynamically interacts with Pyruvate Kinase (PYK4)‐mCherry and their interaction can be measured using FLIM‐FRET. 2‐PGA, 2‐phosphoglycerate; 3‐PGA, 3‐phosphoglycerate; CBC, Calvin–Benson cycle; MPC, Mitochondrial Pyruvate Carrier; OEP, Outer Envelope Protein; PEP, phosphoenolpyruvate; Pyr, pyruvate; TCA, tricarboxylic acid; TPT, triose phosphate transporter; VDAC, Voltage‐Dependent Anion Channel. Created in BioRender.

The tools mentioned above can now be applied to specific systems in which organelle positioning is hypothesized to directly impact metabolic flux. To resolve the endogenous mechanism(s) and significance of the physical linkage between chloroplasts and mitochondria by glycolytic enzyme association, methods that enable the assessment of *in vivo* protein–protein interaction provide powerful means to validate physical interaction. Among others, approaches include Bimolecular Fluorescence Complementation multiplexed with Förster Resonance Energy Transfer (BiFC‐FRET). This *in vivo* approach can provide a framework where stabilization of core interactions (through BiFC) can facilitate the detection of additional partners (through FRET). For example, recent work demonstrated that BiFC of mCitrine irreversibly links the metabolon between Phosphoglycerate Mutase and Enolase (Zhang et al., 2020). Given that the Enolase gene sequence can give rise to two different proteins, Enolase and the ATMBP‐1 (Kang et al., 2013), a Met93Leu mutant variant was used to exclusively generate Enolase protein. In this system, FRET can only occur when the Pyruvate Kinase interacts with the complex. The mCitrine and mCherry stabilize the three‐way enzyme association, since the FRET interaction is mildly stabilized using the WW/WP2 helper peptide system (Grünberg et al., 2013) (Figure 4a). The mCitrineNE and mCitrineCE and mCherry need to be subcloned with the related gene for plant expression and BiFC‐FRET as well as co‐sublocalization analysis (Zhang et al., 2020). Moreover, a complementary strategy would be to express the three‐way construct in plant cell culture and assess its response to exogenous glucose alongside poorly metabolized analogs (e.g., 2‐deoxy‐d‐glucose) to characterize the metabolic control of complex stability. This would provide insight into how metabolite availability regulates the association dynamics of the glycolytic enzyme complex. Being aware that any system to measure interaction dynamics will itself affect the interaction characteristics to some extent, approaches such as splitNanoBRET and a three fluorophore FRET system (Glöckner et al., 2022) offer the possibility to assess reversible interactions. While this strategy, strictly resembles a multi‐enzyme complex rather than a metabolon (Sweetlove & Fernie, 2018), a recent sophisticated use of CRISPR/Cas9 provided a strategy through which inducible metabolons can be produced (Mitkas et al., 2022; Zhang & Fernie, 2022). Inducible control over metabolon organization provides a powerful framework for future synthetic biology applications targeting spatial regulation of metabolism in plants and enabling fine‐tuned metabolic engineering.

## EVALUATION OF THE DYNAMIC ASSOCIATION OF THE GLYCOLYTIC METABOLON AND OTHER ROUTES TO MODIFY ORGANELLAR PARTITIONING

To establish the physiological relevance of enzyme associations linking organelles, their behavior must be evaluated under dynamic environmental conditions. Physiological insight into enzyme assembly‐mediated organelle linkage can be gained by examining how the glycolytic bridge responds to environmental perturbations such as: (i) light‐to‐dark transitions (Pyl et al., 2012), (ii) different rates of photorespiration as altered by the different light intensities (Florian et al., 2014), (iii) photoinhibition by high light (Florez‐Sarasa et al., 2016), (iv) restricted photosynthetic activity by low CO_2_ and low temperature (Hendrickson et al., 2004; Timm et al., 2012), (v) specific inhibitions of distinct mitochondria respiratory chain complexes, or (vi) restricted respiration by chilling stress (Ribas‐Carbo et al., 2000). The degree of association under those conditions may in turn be correlated with macroscopic phenotypes such as photosynthetic rates, respiration, and growth.

In the wild type, the glycolytic metabolon exhibited 19‐times higher efficiency (*K*_cat_/*K*_m_) and lower *K*_m_ values compared with the free enzymes (Zhang et al., 2020). Assessing enzyme activities under different environmental conditions may therefore provide clues about the status of the metabolon. Following this rationale, evaluation of the metabolic consequences of organelle association may be evaluated by estimating changes in the cellular redox landscape by measurement of selected redox enzyme activities and of key redox‐active metabolites. In addition, approaches such as measuring glycolytic intermediates and tracing metabolite labelling following ^13^CO_2_ feeding (Szecowka et al., 2013) enable assessment of the physiological consequences of the formation of the metabolon. Traditional non‐aqueous fractionation approaches (Shapiguzov et al., 2019) can be additionally carried out in order to determine how changes in metabolism are propagated at the subcellular level (Dahmani et al., 2023).

The impact of artificial association of the glycolytic enzyme bridge on metabolic and physiological responsiveness of plants to abiotic stress can be assessed using orthogonal approaches. Inducible transgenic Arabidopsis lines expressing the above‐mentioned SpyCatcher and SpyTag at the outer membrane/envelope of mitochondria and chloroplasts provide a strategy to manipulate organelle proximity, enabling comparison with plants expressing the constitutively bound glycolytic enzymes under equivalent physiological conditions. Such systems further allow time‐resolved analysis of how organelle repositioning influences metabolic and physiological processes. These approaches have the potential to clarify whether the intracellular distribution of mitochondria and chloroplasts does have an impact on the CO_2_ efficiency of C3‐mesophyll cells and whether this ability has direct consequences for the efficiency of net photosynthesis. Given current interest in optimizing photosynthesis, particularly in the face of climate change scenarios, targeted positioning of mitochondria and chloroplasts in the cell may provide an additional layer to the approaches currently pursued (Long et al., 2025; Smith et al., 2023; Sonnewald et al., 2020).

As a further readout of the effects of altered organelle (re‐)positioning, biosensor expression cassettes for subcellular monitoring of ATP, NADH/NAD^+^, and NADPH/NADP^+^ can be introduced into selected genetic backgrounds that have modified organelle positioning. Illumination experiments, such as dark–light transitions with setups such as the recently developed on‐stage illumination imaging, provide a powerful framework to resolve these dynamics *in vivo* (Zheng et al., 2025). These imaging signatures can be complemented by analytical quantification of ATP, ADP, AMP, NAD(P)H, NAD(P)^+^ at specific timepoints in whole leaf extracts, and possibly by non‐aqueous fractionation. Multi‐well plate fluorimetry analysis of leaf discs could be complementary to the confocal microscopy analysis for higher throughput and improved statistics. While a range of treatments can be applied in such systems, dedicated setups for automated illumination still await development. Characteristic ATP and NAD(P) redox dynamics have previously been observed in response to several additional external transitions, including hypoxic stress, dark–light transitions, or changes in carbon status by sucrose feeding (Steinbeck et al., 2020; Wagner et al., 2019; Zheng et al., 2025), supporting the concept that organelle repositioning contributes cellular physiological acclimation to a range of external stimuli.

## CONCLUSIONS AND OUTLOOK

This perspective is motivated by the surprising finding that an enzyme–enzyme assembly of lower glycolysis forms an 18 nm bridge (Zhang et al., 2020) linking mitochondria to chloroplasts. It aims to outline routes to elucidate the functional importance of this structure and to obtain conceptual evidence that may apply to metabolic regulation by organelle positioning in more general. The distance between chloroplasts and mitochondria has been estimated to range from about 10 to 80 nm (Scorrano et al., 2019); therefore, although the complex can only bridge a relatively short distance, it may be sufficient at sites of close organelle contact. Alternatively, additional protein partners may remain to be identified that contribute to bridging across larger distances. Given that an affinity purification approach using phosphoglycerate mutase‐GFP (PGAM‐GFP) in plant cell culture (without developed chloroplasts) did not identify any chloroplast membrane proteins, several candidate chloroplast envelope proteins (including the Triose Phosphate/Phosphate Transporter [TPT], Arabidopsis Outer Envelope Proteins 24 [AtOEP24] and 21 [AtOEP21]) were individually tested for interaction with PGAM. Out of those candidates, only PGAM and TPT showed a positive BiFC signal in both Arabidopsis protoplasts and leaves (Zhang et al., 2020). Since TPT is known to reside in the inner envelope (Fischer & Weber, 2017) and given that a 35S‐driven fusion of mCitrineNE and TPT may result in mislocalization, the mechanistic nature of the putative interaction remains unclear. However, the recent identification that OEP21 from garden pea is the main exit pore for triose phosphates in C_3_ plants (Günsel et al., 2023) provides a potential link between the TPT and lower glycolysis. In this vein, it will be important to test mutants of the Arabidopsis homologs of both OEP21 and OEP24 within the context of organellar positioning. Triose phosphates—as the substrate for the three‐enzyme assembly—may play a physiological role in fast tracking the conversion of 3‐phosphoglyceric acid to pyruvate to supply the mitochondrial TCA cycle. As such, the TPT may be crucial for triose phosphate export from the chloroplast. Consistent with this hypothesis, the metabolon was found associated not only with VDAC in the outer mitochondrial membrane but also with the Mitochondrial Pyruvate Carrier complex (most likely bridged through outer membrane proteins), which has recently been demonstrated to be a functional pyruvate transporter in the inner mitochondrial membrane (Le et al., 2021).

The recent realization that the pea homolog of OEP21 is the major protein involved in the transport of triose phosphates across the outer envelope of the chloroplast (Günsel et al., 2023) not only allows filling a critical gap in the working model of the composition of the glycolytic bridge. It also reinvigorates the need to test the hypothesis that the phosphoglycerate mutase–enolase–pyruvate kinase enzyme bridge represents an efficient means to shuttle photoassimilates preferentially to the mitochondria. The experiments we suggest above provide a path to directly test this hypothesis under a range of physiologically relevant conditions using a battery of genetic, molecular biological, biochemical, and cell biological approaches. This route has the potential to add functional and mechanistic insight to the intriguing descriptive finding of the physical linkage of mitochondria and chloroplasts through a glycolytic bridge.

The implications of this research, however, extend far beyond this pathway. Indeed, we hope that it will stimulate research aimed at several different pathways of primary and secondary metabolism and also of different tissues. Meristem tissues will be of major interest with respect to energy metabolism (Caldana et al., 2023). Similarly, specialized tissues, such as the thermogenic spadix of *Arum maculatum* (Barreto et al., 2025), may serve as models for situations of high flux modes. Another question that will be important to address is to whether in plants glycolytic metabolon formation responds to mechanical cues, as recently found to play a role cancer (Fernie et al., 2020; Park et al., 2020). If we are to understand the significance of spatio‐temporal control of cellular metabolism, we clearly need a synthesis with the principles of cell biological organization and dynamics.

## CONFLICT OF INTEREST

The authors declare no conflict of interest.

## Data Availability

Not applicable, as no new data was created in this study.
